# Regenerative anemia identification in cats: Red blood cell indices or morphology, what to use?

**DOI:** 10.14202/vetworld.2024.1591-1595

**Published:** 2024-07-24

**Authors:** Ana Catarina da Silva Teixeira Fernandes, Daniela Maria Carneiro da Silva, Felisbina Luísa Queiroga, Ana C. Silvestre-Ferreira

**Affiliations:** 1 Department of Clinical Biology, School of Life and Environmental Sciences, University of Trás-os-Montes and Alto Douro, Quinta de Prados, Vila Real, Portugal; 2 Laboratory of Animal Health and Food Safety (Segalab), Argivai, Póvoa de Varzim, Portugal; 3 Department of Veterinary Sciences, University of Trás-os-Montes and Alto Douro, Quinta de Prados, Vila Real, Portugal; 4 Animal and Veterinary Research Centre, University of Trás-os-Montes and Alto Douro, Vila Real, Portugal; 5 Associate Laboratory for Animal and Veterinary Science (AL4AnimalS), Quinta de Prados, Vila Real, Portugal

**Keywords:** anemia, cats, red blood cell indices, red blood cell morphology, reticulocyte

## Abstract

**Background and Aim:**

Anemia, a clinical condition characterized by reduced erythrocytes, is often observed in cats. Regeneration indicates that the bone marrow can respond appropriately to anemia. The absolute reticulocyte count is the reference for differentiating regenerative and non-regenerative anemia, while red blood cell (RBC) indices and morphology provide supplementary information. This study aimed to identify anemia types and establish the most reliable RBC indices and morphology methods in agreement with the reference method.

**Materials and Methods:**

One hundred forty-five cases of cat anemia were prospectively classified using two methods: RBC indices and RBC morphology, and subsequently compared with the absolute reticulocyte count.

**Results:**

Based on RBC indices assessment, 27 cases (19%) exhibited regenerative anemia. Based on RBC morphology, 29 (20%) cases were identified as having regenerative anemia. Using the reticulocyte absolute count as a reference method, 34 (23.4%) cases of regenerative anemia were identified. The findings indicated that RBC indices and RBC morphology did not align in evaluating medullary regeneration and that there is a good degree of agreement between RBC morphology assessment and the reticulocyte absolute count in identifying regenerative anemias.

**Conclusion:**

Blood smear analysis of RBC morphology was more dependable for classifying regenerative anemia than RBC indices. Further studies should be conducted with a larger number of animals and that allow the identification of the cause of anemia and the monitoring of the animal.

## Introduction

Anemia in cats can stem from hemorrhage, hemolysis, or reduced red blood cell (RBC) production in bone marrow [1–3]. Due to their shorter erythrocyte lifespan and lower blood volume, cats are more susceptible to anemia than other species. Different types of hemoglobin (HGB) make anemia more tolerable for them [4]. After diagnosis, anemia needs to be categorized based on its duration, severity, and etiology. The diagnosis of anemia is usually based not only on different RBC parameters but also on signalment, historical and physical examination findings, and serum biochemistry [2, 3]. Identifying whether anemia is regenerative or non-regenerative is crucial in determining its cause [1, 4]. Efficient bone marrow response in regenerative anemia rules out causes other than hemolysis or blood loss [1, 5, 6]. If the anemia is non-regenerative, it is likely that bone marrow dysfunction is present, caused by hyperacute RBC loss, destruction, or decreased production [1, 5].

The variety of helpful methods for assessing regeneration, including RBC indices and morphology as well as reticulocyte count, holds significant importance [2, 5]. The absolute reticulocyte count is the most accurate regeneration indicator [5, 7]. Reticulocytes are macrocytic, characterized by a low HGB content and identified with vital stains such as new methylene blue [4]. When the reticulocyte count is unavailable, it is recommended to perform a blood smear review with the analysis of RBC indices on the erythrogram [7]. The ability of RBC indices and RBC morphology to distinguish regenerative anemia from standard methods remains unexplored.

This study assessed the correlation of RBC indices (mean corpuscular volume [MCV] and mean corpuscular hemoglobin concentration [MCHC]) with RBC morphology in distinguishing regenerative from non-regenerative anemia, and compared which method was more reliable compared to the reference method, reticulocyte absolute count.

## Materials and Methods

### Ethical approval and informed consent

The data used are the results of the routine samples sent to the laboratory; no sample was collected for the purpose of the study, so ethical approval was not necessary. The study was conducted in compliance with Portuguese legislation for the protection of animals (Law 113/2013). Segalab is an external reference laboratory that receives biological samples from all over the country and has no contact with or permission to contact owners. Therefore, verbal or written consent is not possible in this type of study, but as stated in the manuscript, the data used were completely anonymous, and the data of the animals and owners were kept absolutely confidential.

### Study period and location

This retrospective study was conducted from October 2022 to April 2023 at the Segalab - Laboratory of Animal Health and Food Safety, in Póvoa de Varzim, Portugal.

### Sampling and laboratory examination

The study included all cats (n = 145) diagnosed with anemia, irrespective of sex, age, and cause of analysis. One mL of whole cat blood in ethylenediaminetetraacetic acid (EDTA) was taken to the laboratory for the study. The ADVIA 120 hematology system (SIEMENS, Ireland) was used to conduct a complete blood count (CBC) after proper sample homogenization for at least 5 min, following the manufacturer’s guidelines. The following hematological parameters were measured: Hematocrit (HCT), MCV, MCHC, and reticulocyte count. Anemia was defined as an HCT equal to or below 31% [8, 9], and severity was classified, based on HCT, as mild (25–30%), moderate (15–25%), or severe (<15%) [8]. Regarding RBC indices, according to the ADVIA 120 Hematology System, reference intervals for cat MCV vary from 39.0 fL to 55.0 fL and MCHC from 30.0 g/dL to 36.0 g/dL [10]. Anemia was categorized as regenerative or non-regenerative based on MCV and MCHC values. Macrocytic hypochromic anemia was classified as regenerative [11] and presented MCV >55.0 fL and MCHC <30.0 g/dL. The RBC morphology (including polychromasia, anisocytosis, acanthocytes, agglutination, elliptocytes, hypochromasia, Heinz body numbers and percentages, nucleated RBCs, macrocytosis, microcytosis, and schistocytes) was examined through a Wright-stained blood smear. Weiss [8] classified anisocytosis as absent, slight (5–8), mild (9–15), moderate (16–20), or severe (≥20) and polychromasia as absent, slight (1–2), mild (3–8), moderate (9–15), or severe (≥15). The presence of mild polychromasia and anisocytosis was suggestive of regenerative anemia. The absolute reticulocyte count was used to classify anemia as non-regenerative (<0.50 × 109/L), weakly regenerative (50–100 × 109/L), moderately regenerative (100–200 × 109/L), or strongly regenerative (>200 × 109/L) according to Tasker [10].

### Statistical analysis

Agreement between the two medullary regeneration methods was confirmed by matching their corresponding MCV and MCHC values in blood smears displaying polychromasia and anisocytosis. We compared the absolute count of reticulocytes with both methods to determine the most trustworthy approach to assessing medullary regeneration.

Cohen’s kappa coefficient [12] was employed to examine the accord between the corresponding sets of measurements. The coefficient reaches its maximum value of 1 when there is complete concurrence between the two measurements. The coefficient value is more negatively impacted by larger discrepancies between the two assessments. Based on the given cut-off points [13], the agreement between pairs of measurements was categorized as poor (≤0.20), reasonable (0.21–0.40), moderate (0.41–0.60), good (0.61–0.80), or excellent (0.81–1).

κ coefficient, sensitivity, and specificity of the RBC indices and morphology method in comparison to the reticulocyte absolute count method were examined. Sensitivity (%) was calculated as the ratio of true positives to the sum of true positives and false negatives. The results were multiplied by 100. Specificity (%) was calculated as the number of true negatives divided by the sum of the number of true negatives and false positives. The results were multiplied by 100. The data were structured utilizing Microsoft Excel 2016 (Microsoft Corporation, Washington, USA). Frequencies and κ values were computed using the Statistical Package for the Social Sciences version 27 for Windows (IBM Corporation, NY, USA).

## Results

**Figure-1:**
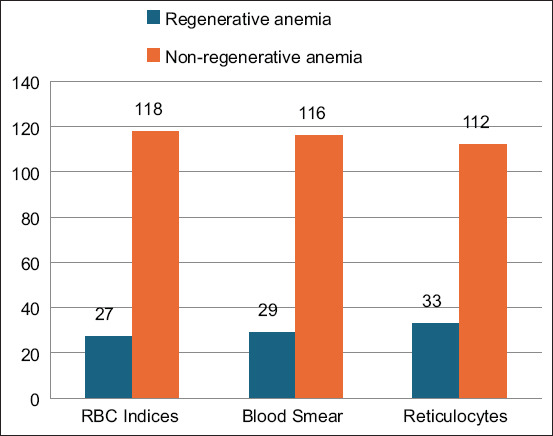
Anemia classification according to the three methods used in the study: RBC indices, RBC morphology, and reticulocyte absolute count. RBC=Red blood cell.

Moderate anemia was found in 85 cases, mild anemia in 42 cases, and severe anemia in 18 cases, constituting 59%, 29%, and 12% of the total cases, respectively. According to the RBC indices assessment, 118 cases (81%) exhibited non-regenerative anemia, while 27 cases (19%) exhibited regenerative anemia (Figure-1).

**Figure-2:**
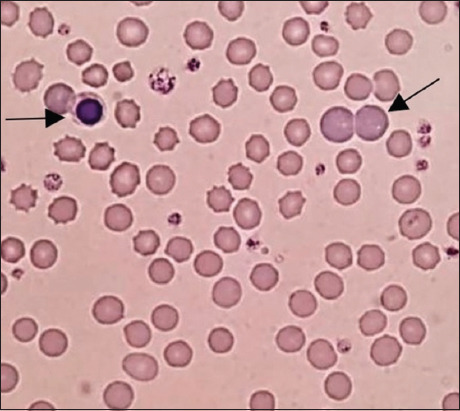
Blood smear from an anemic cat showing moderate polychromasia (visible in the polychromatophilic cells, arrow on the right), anisocytosis (variation in red blood cell [RBC] size), and nucleated RBCs (arrow on the left). These features are representative of regenerative anemia. The platelets were also observed as granular basophilic structures. Wright’s stain, original magnification 1000×.

Based on RBC morphology, 116 (80%) cases were identified as having non-regenerative anemia and 29 (20%) cases as having regenerative anemia (Figure-1). Varying degrees of polychromasia was found in 29 cases; One case with slight, 25 cases with mild, and 3 cases with moderate (Figure-2). Varying degrees of anisocytosis was found in 17 cases, including 4 with slight, 4 with mild, and 4 with moderate anisocytosis, and 5 with severe anisocytosis.

Using the absolute reticulocyte count, 111 cases of non-regenerative anemia (76.6%) and 34 cases of regenerative anemia (23.4%) were identified, among which 15.8% (n = 23) was weakly regenerative, 6.9% (n = 10) was moderately regenerative, and only 0.7% (n = 1) was strongly regenerative.

The comparison of RBC indices and absolute reticulocyte count results for regeneration assessment is shown in Table-1. Using both methods, 96 cases were classified in the same way, but 49 cases were classified differently. The MCV and MCHC methods showed a sensitivity of 22.2% and a specificity of 81.1% compared to the reference method. With a Cohen’s Kappa coefficient of –0.014 (p = 0.868), these two methods cannot be considered concurrent in assessing medullary regeneration.

**Table-1:** Comparison of regeneration assessment between RBC indices and absolute reticulocyte count.

RBC indices	Reticulocytes		Total

	Non-regenerative	Regenerative	
Non-regenerative			
N	90	28	118
Regenerative			
N	21	6	27
Total	111	34	145

N=Number of observations, RBC=Red blood cell

The findings in Table-2 reveal that, based on RBC morphology, 128 cases of anemia showed concordance with the absolute reticulocyte count, whereas 17 cases did not. The RBC morphology method showed a sensitivity of 79.3% and a specificity of 94.6% compared to the reference method. The degree of agreement between RBC morphology assessment and the reticulocyte absolute count as methods for evaluating medullar regeneration was good, with a Cohen’s Kappa value of 0.656, p < 0.001, and percentage of agreement was 88.27%.

**Table-2:** Comparison of regeneration assessment between RBC morphology and absolute reticulocyte count.

RBC morphology	Reticulocytes		Total

	Non-regenerative	Regenerative	
Non-regenerative			
N	105	11	116
Regenerative			
N	6	23	29
Total	111	34	145

N=Number of observations, RBC=Red blood cell

## Discussion

In feline practice, anemia is commonly encountered [1–3]. The bone marrow’s responsiveness determines how anemia should be classified for accurate diagnosis and treatment. Analysis of CBC data and cell morphology serve as crucial methods for anemia classification [7]. Although hematology analyzers have made significant strides in technology, they remain limited in identifying abnormal cells. Erythrogram results need to be validated or contradicted through alternative methods [6, 11].

In this analysis, macrocytosis and hypochromasia were employed as markers for regeneration to categorize anemia as regenerative or non-regenerative [1, 7], with the reticulocyte absolute count as the gold standard. The analyzer and microscope were used to assess these blood cells’ morphological features. Using the ADVIA 120 Hematology System, we calculated the HCT, MCHC, and red cell distribution width (RDW) while the RBC, HGB, MCV, and reticulocyte absolute counts were measured directly. Automated methods analyze a greater number of cells and deliver much quicker results than manual methods. They also eliminate the variability associated with manual methods related to sample staining, dilution, and incubation [14]. Performing a microscopic analysis of a blood smear remains essential for hematological examination. The morphological aspects of blood cells depend on different factors: Conditions of the sample, type of anticoagulant, thickness and drying of the blood smear, coloration method, animal’s age and species, type of cell, and some pathological conditions [15], as well as the operator’s experience recognizing the normal and the abnormal [16]. Analysis of polychromasia and anisocytosis in blood smears was consistently conducted by the same two operators to minimize study result variability.

Analysis of our results revealed a greater incidence of non-regenerative anemia. Evidenced by a high occurrence [17, 18], cats suffer from non-regenerative anemia with an unknown etiology. In a study by Furman *et al*. [19], 57.7% of cats exhibited non-regenerative anemia.

RBC indices and RBC morphology correctly identified the same anemias. The effectiveness of these two methods in distinguishing regenerative from non-regenerative anemia varies. In non-regenerative anemia, we found a greater degree of consensus compared to regenerative anemia. The study’s results showed that the two methods used for medullary regeneration assessment did not align. A study by Samly *et al*. [20] noted discrepancies between the classification of medullar regeneration through RBC indices and peripheral blood smears in human subjects. RBC indices have been previously described as insensitive to correctly identify macrocytic hypochromic anemia, normocytic normochromic (non-regenerative anemia) [11], and microcytic hypochromic anemia (usually iron deficiency anemia) [11, 21].

The absolute reticulocyte count in anemic cats is an accurate method for evaluating medullary regeneration [4, 22, 23]. In our study, reticulocyte counting was done manually or automatically, as done by Tvedten [11]. According to Paltrinieri *et al*. [5], the previously described methods in dogs differ, but Paltrinieri *et al*. [24] reported that both methods yield nearly identical results. Among the sample-related causes for false increases in absolute reticulocyte numbers are Howell-Jolly bodies, Heinz bodies, basophilic stippling, blood parasites, and large platelets with ample RNA [23]. In non-anemic animals, reticulocytosis has been reported, particularly in diseased ones, and is linked to a moderate mortality rate.

Our study identified a lower percentage of non-regenerative anemia using the absolute reticulocyte count classification method compared to the other two. RBC indices can barely discern regenerative anemia compared to the absolute count of reticulocytes and RBC morphology. Furman *et al*. [19] obtained comparable outcomes. MCV and MCHC are not reliable for identifying medullary regeneration. Previous findings have reported comparable outcomes for cats [25], dogs [26], and both [27]. In addition, reticulocyte count and indices added moderate value to the diagnosis of iron-deficiency anemia in cats with chronic kidney disease to the detriment of RBC indices, which were not sensitive to low levels of iron [25].

Regenerative anemia could be identified through RBC morphology. Blood smear analysis can confirm medullary regeneration in cats. A blood smear is a more accurate indicator of medullary regeneration than RBC indices, as previously stated by Hodges and Christopher [7] and DeNicola [22]. Both methods effectively detected non-regenerative anemia, achieving a specificity of approximately 80% based on RBC indices and 95% based on RBC morphology. Previous studies by Furman *et al*. [19] reported similar findings for MCV and MCHC.

The regenerative response to anemia is influenced by factors beyond sampling, including blood collection, transport, and storage. We employed EDTA as the anticoagulant for hematological analysis to ensure optimal preservation of blood cell components and morphology [28]. Prolonged (1 h) incubation of blood samples with EDTA leads to the emergence of artifacts, complicating subsequent analysis of the cells [29]. Analyzing samples at Segalab, which is an external laboratory, took over an hour, potentially introducing more bias [30]. The long storage time of samples can also lead to artifact formation, which interferes with blood smear analysis [31].

## Conclusion

MCV and MCHC do not independently determine anemia’s regenerative or non-regenerative status. Blood smear evaluation of RBC morphology, in conjunction with CBC, is a dependable method for more precise anemia characterization and assessing bone marrow regeneration. However, this retrospective study has a limitation. Only regenerative or non-regenerative anemia was classified, where animals were not closely monitored. There is a need for more comprehensive studies as there is a scope for RBC morphology to provide a better diagnosis and, consequently, treatment of anemia.

## Authors’ Contributions

ACSF and DMCS: Conceptualization. ACSTF and ACSF: Writing – original draft preparation. DMCS and FLQ: Data curation. ACSF and FLQ: Writing – review and editing. ACSTF and DMCS: Methodology. All the authors have read, reviewed, and approved the final manuscript.
